# A case of retropharyngeal emphysema as a complication of pneumothorax

**DOI:** 10.1002/ccr3.3554

**Published:** 2020-11-20

**Authors:** Tsuyoshi Suda, Tomoaki Yoneda, Yukari Ichikawa

**Affiliations:** ^1^ Department of Internal Medicine Kanazawa Municipal Hospital Kanazawa Japan

## Abstract

Retropharyngeal emphysema is a rare condition, and it is important to determine whether the patient presents with complications including pneumomediastinum or other severe clinical presentations such as an upper airway obstruction. In such cases, patients should undergo urgent tracheostomy and surgical neck drainage with concurrent administration of broad‐spectrum antibiotics.

## Graphical abstract

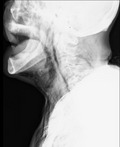

Retropharyngeal emphysema is a rare condition with various causes including spontaneous as well as from traumatic or iatrogenic injuries. Symptoms of mediastinitis and/or airway obstruction should be evaluated as indicators for surgical neck drainage. Complications of mediastinitis are associated with poor prognosis, and patients should be carefully monitored for worsening.

A 73‐year‐old man with idiopathic pulmonary fibrosis developed a left‐sided pneumothorax. The patient complained of dyspnea and mild chest pain. The patient was experiencing hypoxia with an SpO_2_ of 93% under 2 L/min nasal oxygen administration. However, no stridor was observed, and therefore, an upper airway obstruction was ruled out. The patient was afebrile with stable blood pressure and heart rate.

Marked subcutaneous emphysema was observed on the neck and left anterior chest, along with facial disfiguration (Figure 1). Computed tomography revealed retropharyngeal, mediastinal, and subcutaneous emphysema. The emphysema involved the left anterior chest, bilateral neck regions, the surrounding temporal muscles, and the posterior region. Furthermore, pneumomediastinum, extending to the laryngopharynx, and air in the retropharyngeal space were radiographically observed (Figure 2). Based on these results, the patient was diagnosed with retropharyngeal emphysema. Fortunately, he did not show symptoms of airway obstruction. A chest drain was placed for treatment, and drainage was continued. Following drainage, the pneumothorax improved and the retropharyngeal, mediastinal, and subcutaneous emphysema were completely resolved. Retropharyngeal emphysema is a rare clinical disorder with unknown prevalence that can occur spontaneously and may also result from severe maxillofacial injuries or traumatic injuries to the pharynx or esophagus. It can also be caused by iatrogenic factors such as dental procedures or tonsillectomy.^1^ In addition, endoscopic procedures are an important iatrogenic cause of perforations since cervical perforations often occur within the Killian triangle.^2^

**Figure 1:**
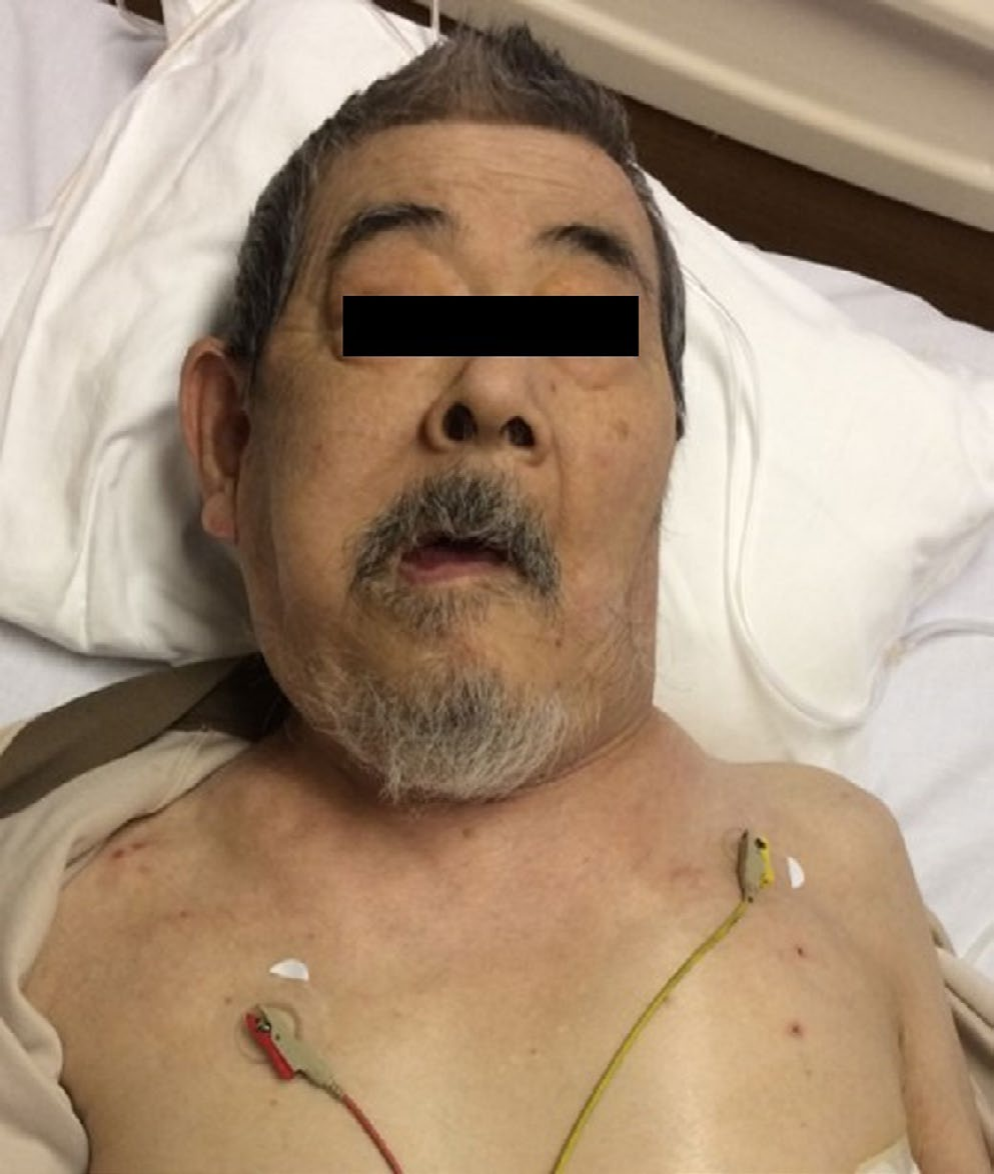
Marked subcutaneous emphysema on the neck and left anterior chest, with facial disfiguration

**Figure 2:**
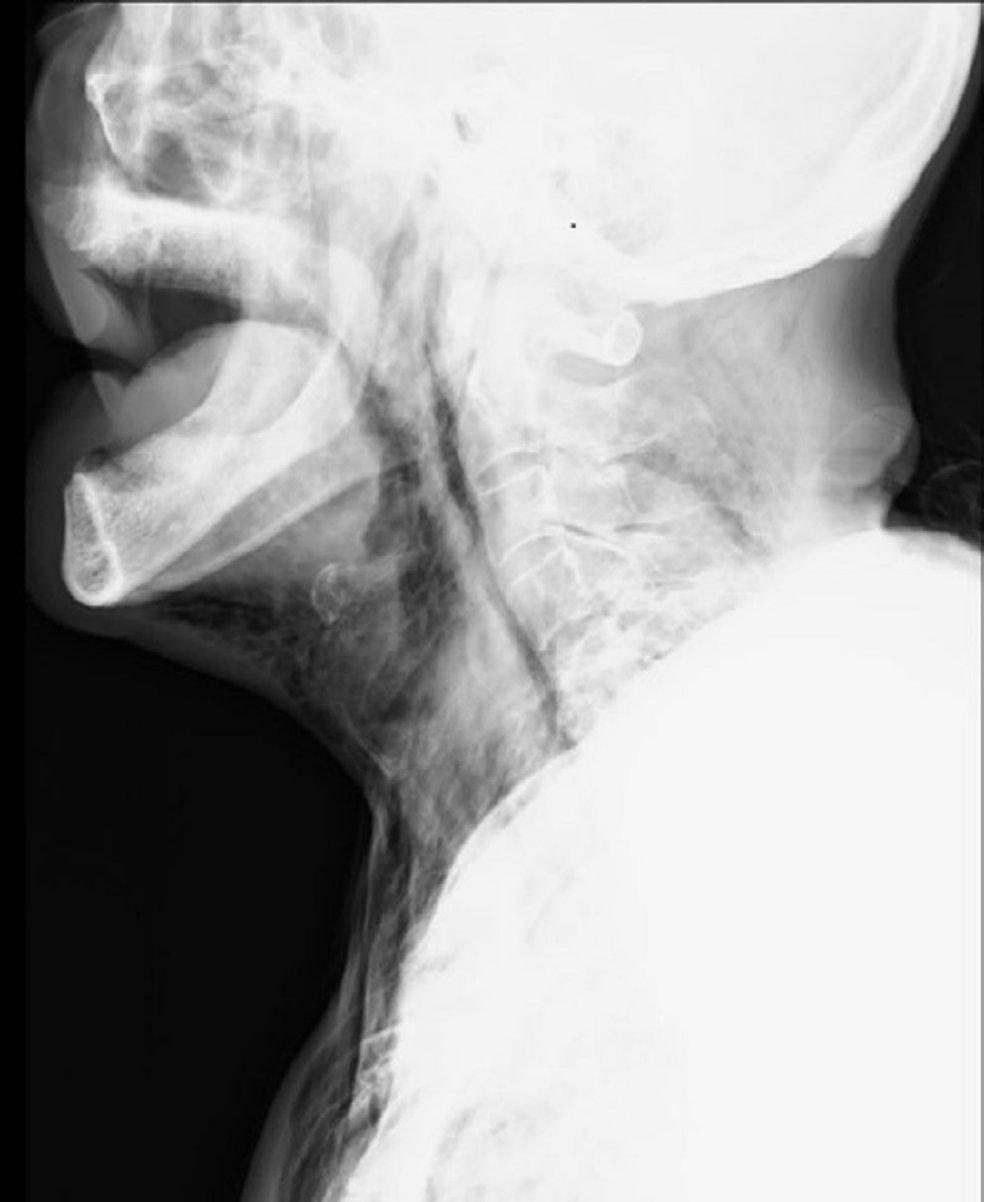
Radiograph of the neck showing pneumomediastinum, extending to the laryngopharynx, and air in the retropharyngeal space

For the treatment and management of retropharyngeal emphysema, isolated patients without attendant pneumomediastinum can be treated conservatively with supplemental oxygen therapy and/or systemic steroid administration and be followed up. For patients that have more severe clinical presentations, especially for cases with upper airway obstructions, treatment should consist of an urgent tracheostomy as well as prophylactic broad‐spectrum antibiotics and should be closely observed. In addition, surgical incision and drainage are often indicated for mediastinitis or symptoms of airway obstruction.^1^ Complications of mediastinitis are associated with poor prognosis.^3^ The appearance of worsening dyspnea, chest pain, abdominal pain, or signs and symptoms related to shock should be carefully monitored.^3^

## CONFLICTS OF INTEREST

There are no conflicts of interest to declare.

## AUTHOR CONTRIBUTION

TS: cared for the patient, conducted the literature search, edited the manuscript, and prepared the figure. TY: contributed to the editing of the manuscript and preparation of the figure. IY: cared for the patient, contributed to the editing of the manuscript, and provided expert opinion on pulmonology.

## Data Availability

The data that support the findings of this study are available on request from the corresponding author. The data are not publicly available due to privacy or ethical restrictions.
